# The Necessities of a Medical School

**Published:** 1900-08

**Authors:** W. W. Keen


### Abstracts and Translations.

### THE NECESSITIES OF A MEDICAL SCHOOL.

BY W. W. KEEN, M.D., LL.D.

[The following brief abstract of the very able address delivered by Dr. Keen, as President, before the American Medical Association, recently held at Atlantic City, contains a forcible statement of facts connected with medical education, and these are equally applicable to dental college work, although in practical training dentistry has long been in advance of medicine.—ED.]

"WHAT were the necessities of a medical school twenty-five years ago? Two lecture-rooms, in which seven professors talked, a dissecting-room, and, if possible, a clinic, which was occasionally, but rarely, in a college hospital. Practically the instruction which the student obtained, with the exception of dissecting, was limited to 'book-knowledge' and 'ear-knowledge.' The student was not brought into contact with any patients or any concrete facts, observations, or experiments. He only listened to what his teachers said about them. Millions were given to hospitals in which the sick were treated, but only sixpences to medical schools, in which the men who were to care for their future patients were trained. 'Spain,' says Lyman Abbott, 'in the late war had nineteenth-century guns and sixteenth-century men behind them. We know what came.' Our splendidly equipped hospitals are the nineteenth-century guns. Insufficiently trained doctors are the sixteenth-century men. The time has certainly come when the 'men behind the guns' must equal in efficiency the weapons with which they do the fighting.

"To perform a tracheotomy and rescue a child suffering from diphtheria is a dramatic occurrence which appeals to every one. To conduct a long series of experiments in the laboratory, by means of which the cause of diphtheria shall be found and the necessity for a tracheotomy avoided, appeals only to the educated few; yet the service done by the operation is a service only to the one patient who may be rescued by the knife, while the other is a service to hundreds and thousands of patients who, for all time, will escape both the knife and the disease. Yet such a series of experiments in preventive medicine brings no reward in money, a limited reward in fame, and only its largest reward in the consciousness of giving a great boon to humanity, for which it never can pay.

"The era of the man who simply listened to what his teachers had to tell him and then went on his way as a 'rule of thumb' man is happily past. This is the era of the trained man and the trained woman, and training means opportunity provided by the community and time, labor, and money given by the man.

"Let us look for a moment at what a medical school now needs. It stands for two things: 1. 'Thing-knowledge' instead of 'book-knowledge' and 'ear-knowledge,' teaching the facts of modern science by scientific methods; that is to say, methods of precision. But, 2, no medical school should be content simply with imparting the knowledge that exists. It should push back the boundaries of ignorance and by research add to our existing knowledge.

"In the accomplishment of the first duty of the medical school there are required, first, didactic lectures. I am not one of those who believe that the day of the didactic lectures is past. 'Never,' said President Faunce, of Brown University, in his notable inaugural, 'never shall we be able to do without the personality of the teacher, flaming with enthusiasm for knowledge, pressing up the heights himself and helping the student on.'

"In the one hundred and fifty-six medical schools in this country there are, perhaps, over fifteen hundred members in their faculties. In all of them are inspiring teachers flaming with enthusiasm, for a not inconsiderable proportion may properly be so described, and the influence of such enthusiastic teachers is felt by the entire class. One or two such men in every school make a good faculty.

"Besides the didactic lectures, a good working library and a reading- or study-room is a requisite. And it is a matter of no little encouragement that in the reports of the United States Commissioner of Education for 1898, seventy-two medical schools reported one hundred and fifty-one thousand four hundred and thirty-three volumes in their libraries.

"The great difference between the modern method of teaching medicine and the older method consists in *laboratory instruction* and *clinical instruction,* both of which must be individual. Laboratories are very costly. They require buildings, equipment, and assistants. The number of laboratories required in the present day in a fully equipped medical school is astonishing. First, the dissecting-room,—the anatomical laboratory,—and along with this a laboratory of histology, and another which may be combined with it, a laboratory of embryology. Next, a physiological laboratory, in which each student will not become an accomplished physiologist, but will become familiar with physiological methods and be trained in exact and careful observation; a laboratory of chemistry and, combined with it, especially, a laboratory of physiological chemistry; in the department of materia medica, a laboratory of pharmacy, where the student will not become a good pharmacist, but will learn the essentials of pharmacy so that he will not make, at least, gross mistakes, which, otherwise, would constantly occur. Still more important is a laboratory of pharmacology, in which he will learn the action of drugs and be prepared rightly to use them. In obstetrics, a laboratory of practical obstetrics and obstetric operations is essential. In surgery, he needs a laboratory in which he shall be taught all the ordinary surgical operations. In pathology, he needs a laboratory of morbid anatomy, a laboratory of bacteriology, and a laboratory of hygiene. The mere statement of this catalogue of thirteen laboratories will enforce the fact that enormous expense not only for the installation, but also for the running expenses, will be required. To show what one university abroad does, Professor Welch has stated [1] that the Prussian government expends, outside of the salaries of professors in the University of Berlin alone, over fifty thousand dollars annually. What American medical school can show anything approaching an endowment which will provide such a sum?

"And what has not the laboratory done for us within the last few years? It has discovered the cause of tuberculosis, tetanus, suppuration, cholera, diphtheria, bubonic plague, typhoid fever, erysipelas, pneumonia, glanders, and a host of other diseases; it has shown us how to avoid all danger from trichina, so that our entire commerce in hog-products is conditioned upon the laboratory; it has shown us how to banish suppuration, erysipelas, tetanus, and pyæmia from our hospitals and reduce our death-rates after operation from fifty or thirty-three per cent. to ten per cent., five per cent., one per cent., and often even fractions of one per cent.; it has given us a really scientific hygiene in which we no longer guess, but know; it has shown us the *rôle* of the mosquito in malaria, of the rat in bubonic plague, of the fly in typhoid fever; it has given us the power to say to diphtheria, 'Thus far shalt thou go and no farther;' it will give us the power to utter a pæan of victory over typhoid, cholera, bubonic plague, tuberculosis, yellow fever, cancer, and other implacable enemies of the human race,—and yet there are those who would stay this God-given hand of help!

"And the laboratory has had not only its devotees, but its heroes. Listen to the story of but one. Dr. Franz Müller, of Vienna, was one of those who in his investigations of the bubonic plague in 1897 contracted the dreaded disease from the bacillus in his culture-tubes. When he became certain that he was infected he immediately locked himself in an isolated room and posted a message on the window-pane, reading thus: 'I am suffering from plague. Please do not send a doctor to me, as, in any event, my end will come in four or five days.' A number of his associates were anxious to attend him, but he refused to admit them and died alone, within the time he predicted. He wrote a farewell letter to his parents, placed it against the window, so it could be copied

[1] "Higher Medical Education and the Need for its Endowment," Medical News, July 21, 1894.

from the outside, and then burned the original with his own hands, fearful lest it might be preserved and carry the mysterious germ. Can you find me a finer example of self-sacrificing altruism? Was ever a Victoria Cross more bravely won?

"But the establishment of laboratories, with their attendant expenses, is not the only improvement in our medical curriculum. Every well-conducted medical school requires a large hospital in connection with it. Here must be installed again a fourteenth laboratory of clinical medicine in which all the excretions of the body will be examined, tumors studied, cultures and blood-counts made, or else the patients in the hospital, from the modern point of view, are neglected. It is not too much to say that a patient requiring such examinations, be he the poorest of the poor, has his case more scientifically studied, more exactly measured, more precisely treated than most rich patients in sumptuous houses.

"Again, the individual instruction to which President Eliot referred is now carried out in all of our best medical-school hospitals by the establishment of small ward-classes, by whom or before whom the patients are examined, prescribed for, and operated upon by the professor or instructor, each student bearing a part; and so, by having his investigations directed, his powers of observation cultivated, his mistakes pointed out, his merits applauded, the student graduates from the medical school equipped as none of us, alas! ever had the opportunity to be. All of these laboratory and ward-classes imply an enormous increase in the number of assistants, young men striving not only to perfect themselves, but, by teaching, to forge to the front so that the best men will win in the struggle for preferment.

"Again, the course of study has been prolonged from two years, as it was twenty to twenty-five years ago, to four years, and in addition the terms have also been lengthened. When I was a student the course of study consisted of two sessions of about nineteen weeks each, or thirty-eight weeks in all. Now the course consists, as a rule, of four sessions of thirty-two weeks each, or a total of one hundred and twenty-eight weeks, an increase of ninety weeks, nearly three and one-half times as much as it was twenty-five years ago. In 1885, one hundred and three schools had courses of two years, and five schools courses of three years. In 1899, two schools had courses of two years, ten of three years, and one hundred and forty-one of four years.

"It can be easily seen that from this additional time required another source of expense has arisen besides the increased number of assistants. The time given to teaching by members of the faculty, as a rule, has been more than tripled, as compared with twenty-five years ago. In addition to this, professors in charge of laboratories must practically give their whole time to the work, and are precluded, therefore, from any income from practice. These men must receive salaries sufficient for them to live on.

"Surely this statement of the difference between the education given twenty-five years ago, which required but little expenditure of money and resulted in considerable incomes, and the modern methods of education in the laboratory and the hospital, as well as the lecture-room, which require enormous expenses, is an ample reason for large endowments."—*Journal of the American Medical Association.*

